# The global impact of the DRACMA guidelines cow’s milk allergy clinical practice

**DOI:** 10.1186/s40413-017-0179-7

**Published:** 2018-01-04

**Authors:** Alessandro Fiocchi, Holger Schunemann, Ignacio Ansotegui, Amal Assa’ad, Sami Bahna, Roberto Berni Canani, Martin Bozzola, Lamia Dahdah, Christophe Dupont, Motohiro Ebisawa, Elena Galli, Haiqi Li, Rose Kamenwa, Gideon Lack, Alberto Martelli, Ruby Pawankar, Maria Said, Mario Sánchez-Borges, Hugh Sampson, Raanan Shamir, Jonathan Spergel, Luigi Terracciano, Yvan Vandenplas, Carina Venter, Susan Waserman, Gary Wong, Jan Brozek

**Affiliations:** 1 Department of Clinical Epidemiology & Biostatistics, McMaster University Health Sciences Centre, 1200 Main Street West, Hamilton, ON L8N 3Z5 Canada; 2 Department of Allergy & Immunology, Hospital Quironsalud Bizkaia, Carretera Leioa-Unbe 33 bis, 48950 Erandio - Bilbao, Spain; 3 Division of Allergy and Immunology, Cincinnati Children’s Hospital Medical Center, Cincinnati, OH USA; 4 Pediatrics & Medicine, Allergy & Immunology, Louisiana State University Health Sciences Center, Shreveport, LA USA; 5 Department of Translational Medical Science, European Laboratory for Investigation of Food Induced Diseases and CEINGE Advanced Biotechnology, University of Naples Federico II, Naples, Italy; 6 Department of Pediatrics, British Hospital, Perdriel 74, CABA, Buenos Aires, Argentina; 7 Department of Pediatric Gastroenterology Hepatology and Nutrition, Hôpital Necker Enfants Malades, Paris, France; 8 Department of Allergy, Clinical Research Center for Allergy and Rheumatology, Sagamihara National Hospital, Sagamihara, Kanagawa Japan; 9 Pediatric Allergy Unit, Research Center, San Pietro Hospital - Fatebenefratelli, Rome, Italy; 10 Pediatric Division, Department of Primary Child Care, Children’s Hospital, Chongqing Medical University, Chongqing, China; 11 Department of Pediatrics and Child Health, Aga Khan University Hospital, Nairobi, Kenya; 12 King’s College London, Asthma-UK Centre in Allergic Mechanisms of Asthma, Department of Paediatric Allergy, St Thomas’ Hospital, London, UK; 13 Department of Pediatrics, Salvini Hospital, Milan, Italy; 14 Department of Otolaryngology, Nippon Medical School, 1-1-5 Sendagi, Tokyo, 113 Japan; 15 Allergy & Anaphylaxis Australia (A&AA) organisation, Sydney, Australia; 16 Department of Allergy and Clinical Immunology Centro Médico-Docente La Trinidad Caracas, Caracas, Venezuela; 17 Department of Pediatrics, Jaffe Food Allergy Institute, New York, USA; 18 Icahn School of Medicine at Mount Sinai, Box 1089, New York, USA; 19 Institute of Gastroenterology, Nutrition and Liver Disease, Schneider Children’s Medical Center, Sackler Faculty of Medicine, Tel-Aviv University, Tel-Aviv, Israel; 20 Division of Allergy and Immunology, Department of Pediatrics, The Children’s Hospital of Philadelphia, Perelman School of Medicine at University of Pennsylvania, Philadelphia, PA USA; 21 National Pediatric Healthcare System, Board member of the Italian Pediatric Respiratory Society, ATS, Milan, Italy; 22 Department of Pediatrics, UZ Brussel, Vrije Universiteit Brussel, Brussels, Belgium; 23 Section of Allergy & Immunology, University of Colorado Denver School of Medicine | Children’s Hospital Colorado, Aurora, CO USA; 24 Department of Medicine, Clinical Immunology and Allergy, McMaster University, Hamilton, ON Canada; 25 Department of Paediatrics, Faculty of Medicine, The Chinese University of Hong Kong, Sha Tin, Hong Kong; 26 Division of Allergy, Department of Pediatrics, Pediatric Hospital Bambino Gesù, Piazza Sant’Onofrio, Vatican City, Rome Italy

## Abstract

**Background:**

The 2010 Diagnosis and Rationale for Action against Cow’s Milk Allergy (DRACMA) guidelines are the only Grading of Recommendations Assessment, Development and Evaluation (GRADE) guidelines for cow’s milk allergy (CMA). They indicate oral food challenge (OFC) as the reference test for diagnosis, and suggest the choice of specific alternative formula in different clinical conditions. Their recommendations are flexible, both in diagnosis and in treatment.

**Objectives & methods:**

Using the Scopus citation records, we evaluated the influence of the DRACMA guidelines on milk allergy literature. We also reviewed their impact on successive food allergy and CMA guidelines at national and international level. We describe some economic consequences of their application.

**Results:**

DRACMA are the most cited CMA guidelines, and the second cited guidelines on food allergy. Many subsequent guidelines took stock of DRACMA’s metanalyses adapting recommendations to the local context. Some of these chose not to consider OFC as an absolute requirement for the diagnosis of CMA. Studies on their implementation show that in this case, the treatment costs may increase and there is a risk of overdiagnosis. Interestingly, we observed a reduction in the cost of alternative formulas following the publication of the DRACMA guidelines.

**Conclusions:**

DRACMA reconciled international differences in the diagnosis and management of CMA. They promoted a cultural debate, improved clinician’s knowledge of CMA, improved the quality of diagnosis and care, reduced inappropriate practices, fostered the efficient use of resources, empowered patients, and influenced some public policies. The accruing evidence on diagnosis and treatment of CMA necessitates their update in the near future.

## Background

The mission of the World Allergy Organization (WAO) is to advance excellence in clinical care, research, education and training. Clinical practice guidelines are part of this mission. In the last 10 years, WAO produced guidelines on anaphylaxis, allergy prevention, urticaria, allergy training, hereditary angioedema, and molecular diagnosis [1]. All of these documents aim at deepening the clinician’s knowledge, improving the quality of diagnosis and care and reducing inappropriate variation in practice. Application of these guidelines may promote the efficient use of resources, inform and empower patients and support public policies [2]. However, their introduction into routine daily practice requires a series of educational, social and political steps. If not correctly implemented, the guidelines may fail their objectives and patients may remain exposed to harmful or unnecessary care [3]. Barriers to guideline implementation may be encountered at different levels [4]:

- Individual, as professionals may have difficulty understanding the guidelines’ language; they may also introduce personal bias in thinking, balancing benefits and risks, and reaching different conclusions;
- Motivational, as different factors/barriers may generate different motivational stages in individual professionals;
- Relating to organizational context, for instance lack of arrangements for continuous learning, and lack of implementation tools;
- Social, for the interference of existing values and cultures, and for the influence of the opinion of key people;
- Economic, for insufficient or no reimbursement arrangements, rewards, health care systems or incentives.

To overcome these limitations, a series of educational tools needs to be put into play. In this article, we will evaluate the impact on real life of DRACMA, the GRADE guidelines on diagnosis and treatment of CMA [5], and their dissemination.

### DRACMA’s influence on the subsequent literature

The original version, published in the World Allergy Organization Journal (WAO Journal), was co-published in Pediatric Allergy and Immunology (PAI) [6]. In 2011, DRACMA was the most downloaded article from PAI website, the second in 2012 and the third in 2013. The publication in the WAO Journal was the most accessed article in 2011 and 2012. The last available data (up to 2015) still indicate that it ranks in the top ten. Up to August 15, 2017, according the Scopus data, 241 articles cited the two versions. A summary report was published at the end of 2010 [7]. As for mid-august 2017, it has been cited 109 times thus far, with a 6.57 Field-Weighted Citation Impact. The systematic review proposing the recommendations for Oral Immunotherapy in CMA [8] has been cited 103 times with a 6.12 Field-Weighted Citation Impact. Thus, DRACMA influenced heavily the subsequent literature on CMA.

### DRACMA publications

After ARIA (Allergic Rhinitis and its Impact on Asthma), DRACMA was the second guideline in allergy medicine focused on important patient outcomes, explicitly taking into consideration the patient’s values and preferences. It pioneered in applying a systematic approach to collecting the evidence, to separate the concepts of quality of evidence and strength of recommendations, and to transparently report the decision process. The method used for this CMA guideline was highlighted as an example of application of the GRADE methodology in an article cited 58 times [9]. The application of such principles to the diagnostic tests for CMA warranted a specific report, which has been cited 42 times [10].

Other articles reported on the global burden of CMA [11], and on its clinical aspects after the publication of the guideline [12–14].

### Guidelines on diagnosis and treatment of food allergy before and after DRACMA

Prior to DRACMA, a handful of guideline documents for food allergy diagnosis and treatment had been issued by the main scientific societies in America and Europe [15–17]. National position papers and guidelines were available in the Netherlands [18], Finland [19], Spain [20], France [21], Germany [22] and Japan [23]. In general, these guidelines were intended for specific countries and/or for specific geographical areas, so they took stock of local factors of epidemiological, economic, organizational, and social nature. None of these documents used the GRADE methodology.

After 2010, other guidelines in the field of food allergy were proposed. One of them made use of the GRADE methodology in a way similar to DRACMA [24], another used some form of GRADE [25], and others were consensus-based documents [26–30]. Some national guidelines were also updated or issued [31–34]. During its 7 years, DRACMA was compared to other food allergy guidelines, illustrating how the values and preferences expressed by the writing committees can modify the recommendations [35, 36].

The number of citations may reflect the relevance of the different food allergy guidelines: the most cited is the National Institute of Allergy and Infectious Diseases (NIAID) guideline [24] (392 citations, 5.17 Field-Weighted Citation Impact). DRACMA stands second (241, 6.26), followed by the European Academy of Allergy and Clinical Immunology (EAACI) guidelines [25] (210, 18.68 Field-Weighted Citation Impact).

### Guidelines on diagnosis and treatment of CMA before and after DRACMA

By 2010, a few consensus documents provided guidance on the diagnostic and therapeutic aspects of CMA in children [37, 38]. National position papers and guidelines had been produced in Germany [39, 40], Italy [41] and Argentina [42], reflecting general and local needs and vision.

After the publication, 93 WAO-affiliated national Allergy Societies endorsed the DRACMA guideline. Many of the national meetings of these societies hosted lectures on the topic. DRACMA was presented in many countries, in US, France, Italy, Brazil, Chile, Argentina, Kenya, Egypt, Thailand, and Indonesia, to name a few. In addition, some Allergy Societies outside of WAO, e.g. the Iranian, invited WAO lecturers to present on DRACMA. Following the DRACMA explicit invitations to national implementation, some scientific and regulatory bodies did discuss and actualize it in France [43, 44], United Kingdom [45, 46], Middle East [47], South Africa [29, 30]. In Mexico, the DRACMA recommendations were incorporated in a large specific guideline [48].

In other cases, the DRACMA guidelines were directly translated into the national languages, to overcome language barriers. This happened in Italy [49], in South America with the Spanish translation [50] and in China [51]. The Mandarin translation was also discussed to be actualized in the Chinese context [52].

After these discussions in many countries, DRACMA is now the most cited CMA guideline, followed by the European Society for Paediatric Gastroenterology Hepatology and Nutrition (ESPGHAN) guideline on cow’s-milk protein allergy [53] (179 citations, 16.56 Field-Weighted Citation Impact) and by the Italian CMA guideline [41] (51 citations, 4.02 Field-Weighted Citation Impact). The British Society of Allergy and Clinical Immunology (BSACI) guidelines [46] score 4th (42 citations, 11.57 Field-Weighted Citation Impact).

### Economic consequences of DRACMA: Diagnosis

**Fig. 1:**
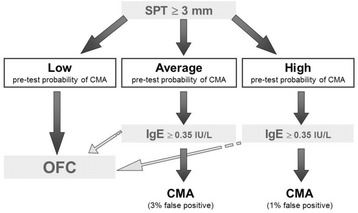
In settings where OFC is not considered a requirement, should in vitro specific IgE determination be used for the diagnosis of CMA in patients suspected of CMA and a positive result of a skin prick test? [13]

**Fig. 2:**
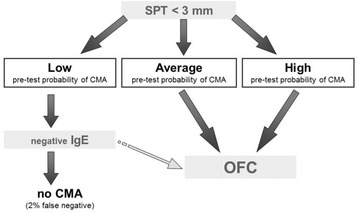
In settings where OFC is not considered a requirement, should in vitro specific IgE determination be used for the diagnosis of CMA in patients suspected of CMA and a negative result of a skin prick test? [13]

Among the diagnostic approaches proposed during the phases of national adaptation, the British example is of particular interest. In DRACMA, metanalyses of the available literature allowed us to calculate the performance characteristics of common diagnostic methods (skin prick test [SPT] and specific IgE determination, at the cut-off values of 3 mm wheal diameter and 0.35 kU_A_/L respectively) vs. the Oral Food Challenge (OFC) reference test. Assessing the clinical history, physicians can determine the diagnostic likelihoods estimating the pre-test probability of CMA. As examples, the pre-test probability will be low in cases of atopic dermatitis or Gastroesophageal Reflux Disease (GERD), average in case of immediate reactions or high in case of anaphylaxis. The DRACMA guidelines recommend – when possible – OFC for diagnosing CMA, to avoid the risk of anaphylactic reactions at home in SPT or sIgE false negative cases, unnecessary treatment for false positive cases and inappropriate resource utilization. However, some reasons (availability of appropriate staff, organizational obstacles, resource availability, etc.) may make it difficult to perform an OFC. In settings where OFC is not considered possible or opportune, a pre-test probability estimate may help physicians to reach a highly probable diagnosis using simpler diagnostic tests such as SPTs and/or specific IgE determination. These diagnostic pathways however, allow a small chance of false positive or negative results (Figs. 1 and 2) [13].

The cost of challenge test is reasonable in the majority of cases. In the British context however, challenges were considered "time-consuming and expensive" [46]. For this reason, the BSACI guidelines indicated UK as a setting where OFC is not considered an absolute requirement for the diagnosis of CMA. Taking stock of the DRACMA assessment of the probability of false-positive and false-negative diagnosis in case of high- medium- and low- pretest probability, they recommended the use of history (“typical” vs “non-typical”) and SPT as rule-out and diagnostic tests in clinical practice at the primary level. Especially for non IgE-mediated CMA, they underline the role of dietary elimination for the diagnosis. This approach, limiting the role of milk challenge to most doubtful cases, is similar to that proposed by the ESPGHAN "practical" guideline, issued in 2012 [53]. This choice is perhaps cost-effective, but may expose patients to the risk of overdiagnosis. As an example, in the Northern-Irish experience, the application of such strategy resulted in a reduction of prescriptions for symptomatic drugs for GERD, but in a steady increase in prescriptions for special formulas [54]. Although one may surmise that the diagnostic costs are reduced, the net costs for CMA treatment increased in that community [55]. This example illustrates how the application of a guideline can influence real life practices and economics.

### Economic consequences of DRACMA: Treatment

The DRACMA recommendations proposed an appropriate substitute for different clinical situations. The question on substitute formulas was the following: "Should amino acid formula, extensively hydrolyzed whey or casein formula, soy formula or rice formula be used in children with IgE-mediated CMA?".

The answer to this clinical question was structured through the recommendations in the box.

#### Box: DRACMA recommendations for CMA management

***Recommendation 7.1***

*In children with IgE-mediated CMA at high risk of anaphylactic reactions (prior history of anaphylaxis and currently not using extensively hydrolyzed milk formula), we suggest amino acid formula rather than extensively hydrolyzed milk formula (conditional recommendation/very low quality evidence).*

***Underlying values and preferences***

*This recommendation places a relatively high value on avoiding possible anaphylactic reactions and a lower value on avoiding the direct cost of amino acid formula in settings where the cost of amino acid formulas is high.*

***Remarks***

*In controlled settings, a trial feeding with an extensively hydrolyzed milk formula may be appropriate.*

***Recommendation 7.2***

*In children with IgE-mediated CMA at low risk of anaphylactic reactions, (no prior history of anaphylaxis or currently on extensively hydrolyzed milk formula), we suggest extensively hydrolyzed milk formula over amino acid formula (conditional recommendation/very-low quality evidence).*

***Underlying values and preferences***

*This recommendation places a relatively high value on avoiding the direct cost of amino acid formula in settings where the cost of amino acid formula is high. In settings where the cost of amino acid formula is lower, the use of amino acid formula may be equally reasonable.*

***Remarks***

*Extensively hydrolyzed milk formula should be tested in clinical studies before being used. If a new formula is introduced, one should carefully monitor if any adverse reactions develop after first administration.*

In structuring these recommendations, formulas were rated according to a series of parameters. Among them, the price was explicitly indicated as an important factor. The DRACMA panel did a preliminary survey of the mean cost of different types of formulas worldwide (Table 1), from which it was found that feeding an infant with an extensively hydrolyzed formula (eHF) was 2.5 times less expensive than using an amino acid-based formula (AAF). Thus, even if the safety of AAF was higher than eHF, the latter was indicated as the first choice in CMA, except in cases of severe forms CMA with high reactivity (anaphylaxis or eosinophilic esophagitis), where AAF was recommended. Soy formulas (SF) were considered less useful to avoid reactions to soy unless they were more available and negative to skin testing. Extensively hydrolyzed rice formula (eHRF) is probably safer than eHFs, but it was considered at a lower level because it is not present in many countries (including UK).

**Table 1:** Mean cost of special formulae worldwide, assessed in October 2009 and used in DRACMA Guidelines, vs. price structure in Italy after the DRACMA implementation [5]

Formula	Cost (€/liter)	Cost (€ per month)	Cost (€/liter)	Cost (€ per month)
Cow’s milk	0.9	20	1.50	30
Cow’s milk formula	2.0	45	2.0	44
Soy formula	5	112	6	132
eHF	6.5	135	6.3	139
eHRF	6	135	7.5	165
AAF	14	318	12.8	281

As every recommendation reports the outcome that was considered most relevant by the expert panel (Box 1), they are flexible and can be subject to different interpretations when the importance of the outcomes in a particular country, or for a particular patient, is different. As the cost of the same formula differs substantially from country to country [56], the implementation of the recommendations may differ.

In recommendations 7.1 and 7.2, for example, cost makes AAF a second choice when the clinical risk is lower (see “values and preferences”). Elaborating on these considerations, an Italian company decided in 2012 to decrease the cost of their AAF by 30%, so that the cost of AAF dropped from 2.4 to 2 times that of eHF. This did modify the balance of recommendations for a substitute formula. AAF were proposed to children with even less severe forms of CMA, such as CM protein-induced atopic dermatitis.

This example illustrates how DRACMA guidelines did influence the formula market, making appropriate treatments affordable to larger layers of population. Naturally, this is only one of the factors for an appropriate care. In some countries, patients are reimbursed for AAF if “allergy” to eHF has been demonstrated, in others there are no reimbursement policies. This can expose to over-or under-use of special formulas.

## Conclusions

DRACMA promoted a cultural debate among researchers and clinicians, improving the quality of diagnosis and clinical care. The accruing evidence on diagnosis and treatment supports the need for an update. Ideally, the new DRACMA guidelines should include non IgE-mediated CMA, particularly mild-moderate forms of CMA and chronic FPIES, as this part of the discipline has never been subjected to the strictest criteria for EBM, using the GRADE approach. We envisage the updated DRACMA will answer more clinical questions, serving the patients’ and the pediatricians’ needs in the various contexts.
